# Pulmonary hypertension: From an orphan disease to a global epidemic

**DOI:** 10.21542/gcsp.2020.5

**Published:** 2020-04-30

**Authors:** Ghazwan Butrous

**Affiliations:** Medway School of Pharmacy University of Kent at Canterbury, UK

## Introduction

Pulmonary hypertension is a progressive disease characterized by an elevation of pulmonary artery pressure and pulmonary vascular resistance, leading to right ventricular failure and death. It remains a challenging chronic progressive disease, but the current interest and advent of medical therapy in the last 20 years has significantly changed the perception of medical community in this disease^1–3^. Pulmonary hypertension is not a specific disease; the majority of cases present with other diseases and various pathological processes that affect the pulmonary vasculature, and consequently increase pulmonary pressure and vascular resistance^3,4^.

It has been considered a rare “orphan disease”, but now many more patients are diagnosed and managed appropriately^5,6^. Thus, the question I try to reflect and deliberate is if this condition can still be considered and orphan (rare) disease, or our increase in interest and awareness can lead us to discover more patients globally^4^. I shall start with the current definition of orphan diseases, and then progress to the historical medical background of how we have developed our understanding about this condition in the twenty-first century. Later we shall try to investigate the “epidemiological” pattern of this pulmonary hypertension as we infer it today.

## The definition of orphan/rare disease

An orphan disease is broadly defined as a condition that affects a small number of patients. Unfortunately, no easy or single definition is widely accepted^7^. In the USA, if a disease affects fewer than 200,000 people in the country, it is defined as “orphan disease”^8,9^. The European definition is a disease that occurs with a prevalence of less than 1 in 2,000^10^. Other definitions add conditions that are often complex and may be challenging to diagnose correctly, or maybe misdiagnosed in up to 36% of patients^11,12^.

There are currently around 7000–8000 diseases, affecting 400 million people globally, that can be defined as “orphan diseases”^13,14^. The majority are due to genetic defects with different clinical phenotypes^8,10^. The advancement of genetic research is now significantly contributing to more additions to this category of diseases^7,15,16^.

Some in the western world consider diseases prevalent in poor developing countries (such as tuberculosis, cholera, typhoid, and malaria) in the category of orphan diseases because the return of investment in drug development is low^17^. The main drivers to this definition are economical, which attracted the attention of the policymakers, health authorities as well as drug developers and pharmaceutical industry.

The development of drugs against orphan diseases was not attractive to the pharmaceutical industry because there was little financial incentive. Thus, SA legislators passed the “Orphan Drug Act of 1983” to encourage more R&D in this category of diseases. The act was amended in 2002^9,18^, which facilitated the establishment of the Office of Rare Diseases as a federal entity. This entity was able to recommend a national research agenda, coordinate research, and provide educational activities for researchers.

As a result of these legal initiatives, many programs at the FDA and the NIH began encouraging product development, as well as clinical research for products targeting rare diseases^19^. Many other countries, like Japan in 1993 and the European Union in 2000, adopted the same legal framework^20^.

The interest in orphan/rare diseases is not abating and many laws and initiatives have been introduced to deal with these conditions^10,12^ such as Orphanet^21^ and the EC Expert Group on Rare Diseases^22^ in Europe. In the United States, similar organization are the National Organization for Rare Disorders (NORD)^23^, the Office of Rare Disease Research (ORDR)^24^, as well as the Food and Drugs Administration (FDA )^25^. There are even specialized journals like Orphanet Journal of Rare Diseases^26^.

## Increasing interest in pulmonary hypertension^27–29^

In 1891, Ernst von Romberg, a German physician, described an autopsy with “sclerotic” changes in the arteries of the lungs^30^. This report was the first to attract the attention of other investigators to describe the pathology of the pulmonary vasculature in various other clinical conditions.

Dr Abel Ayerza from Argentina in 1901, described a 38-year-old male patient with a history of cough, dyspnea, severe cyanosis, and manifestations of right heart failure. The patient died 24 days after admission. The autopsy showed cardiomegaly due to thickening of the right ventricular wall and right atrium dilatation.

He described dilated and thickened wall bronchi full of secretions. The pulmonary arteries showed thrombus obstructing the lumen and hyperplasia of both middle and intima layers, with new glomerular-like “formed channels” blocking the pulmonary vessels^31^. Dr Ayerza called it “*cardíaco negro*” (black heart), due to the extreme cyanosis observed in this patient.

Following this detailed description, several more similar cases of the “black heart” were published by Dr Pedro Escudero in 1905 and 1911, who considered it secondary to a chronic pulmonary process and the comorbidities between syphilis and obliterating sclerosis of the pulmonary artery^32^.

Dr F.C. Arrillaga, a student of Dr Ayerza, described a further 11 patients and proposed that injury of the pulmonary artery was secondary to chronic lung processes and would cause right heart hypertrophy^33^. Thus, at the beginning of the 20th century, pulmonary hypertension became known as “Ayerza’s disease”.

The common belief that syphilis was the leading cause of pulmonary hypertension persisted until 1935, but was later challenged in the 1940s, when Dr Oscar Brenner^34,35^ described the histopathological changes of 100 patients with pulmonary hypertension.

The second milestone in the history of pulmonary hypertension was after the introduction of the cardiac catheterization technique in 1930-1940s. This technique was inspired by a German physician, Werner Forssman, who had catheterized himself in 1929 via the antecubital vein and published a picture of the catheter in his heart^36^.

Dickinson W. Richards and Andre F. Cournand later refined the technique of right-heart catheterization^28,37^. This achievement awarded the three pioneers the Nobel Prize of Physiology and Medicine in 1956. Many Cournand and Richard fellows, in particular David Dresdale, described an increase in pulmonary pressure in patients with pulmonary hypertension using cardiac catheterization;^38^. Some of Dresdale’s cases were with no apparent cause and were labelled as “primary pulmonary hypertension”^38^.

Paul Wood, in 1958, confirmed these findings using cardiac catheterization. He described the concept of reactive pulmonary hypertension, which differed in various conditions for the first time^39^. Alfred Fishman, who was also a fellow of Cournand and Richard, elaborated in 1961 on the different pathophysiological changes of pulmonary hypertension^40^.

These developments led to study of pulmonary vasculopathy in congenital heart disease by Dr. Vic Harrison at James Henry Dible’s pathology lab at the Royal Postgraduate Medical School of London. He encouraged his new newly-arrived Dutch fellow, Dr C. Wagenvoort and his wife Mrs Noek Wagenvoort, to study pulmonary vascular pathology, which became a lifelong focus of their research on the pathological features of pulmonary hypertension around the world^41^.

In 1970, they published the most extensive series of autopsies of patients (156 cases, of which 105 were females) collected from 51 centres worldwide^42^. They reported arteries with medial layer hypertrophy, laminar intimal fibrosis, and fibrinoid necrosis, inflammatory cells and plexiform lesions. Their series included, according to their classification, 110 patients with ”primary pulmonary hypertension”, 31 with chronic thromboembolism, five were described as having chronic pulmonary venous hypertension, 5 with pulmonary veno-occlusive disease, one with sarcoidosis, one with pulmonary schistosomiasis, and 3 with chronic bronchitis and emphysema.

They later divided pulmonary hypertension into five groups:

• Group 1. Pulmonary hypertension due to tricuspid valve insufficiency or post -tricuspid shunts.
• Group 2. Pulmonary hypertension due to chronic pulmonary embolism.
• Group 3. Pulmonary hypertension due to obstructed pulmonary venous outflow.
• Group 4. Pulmonary hypertension due to chronic respiratory disease and hypoxia.
• Group 5. Decreased pulmonary vascular flow^43,44^.

The third milestone in the history was the development of a pulmonary hypertension epidemic in Austria, the Federal Republic of Germany, and Switzerland, starting in the late 1960s. There was a significant increase in the number of patients with pulmonary hypertension due to the appetite suppressant aminorex^45^ (see ‘Appendix’).

This epidemic led the World Health Organization (WHO) to hold a meeting in Geneva in October 1973 to assess the significance of pulmonary hypertension as a rare disease. Shuichi Hatano and Toma Strasser edited and published the final report in1975^46^. The report emphasized the rarity of pulmonary hypertension and the epidemic that showed a sudden increase in the number of patients.

The attendees defined pulmonary hypertension as a mean pulmonary pressure of 25 mm Hg and suggested that more studies are needed to evaluate the effect of exercise on hemodynamics. It distinguished precapillary hypertension (that includes primary pulmonary hypertension) from postcapillary hypertension. They also identified other conditions that were reported to cause pulmonary hypertension, specifically hypoxia, altitude, drug-related PH, recurrent thromboembolism, congenital heart disease, and chronic lung disease (such as emphysema).

The report also acknowledged the familiar forms of the disorders (referring to the five publications reporting 47 cases in 18 families). The works of Wagenvoort and Wagenvoort^42^ were adopted in the pathological grading of arteriopathy and the presence of specific lesions.

Dr C A Wagenvoort and Dr Donald Heath suggested using the term “plexogenic pulmonary hypertension” when the morphological entity characterized by concentric-laminar intimal fibrosis, fibrinoid necrosis, and plexiform lesions. The term ”primary pulmonary hypertension” was ascribed to unexplained pulmonary hypertension^41^. Although this can be considered reasonable, it proved to be challenging to implement clinically as histopathology is not always available to the treating physician^41^.

The Geneva meeting concluded by encouraging more collaborative work and strongly recommended an international registry on pulmonary hypertension. Subsequently, in 1981, Alfred Fishman led the efforts to establish this registry, that was funded by The National Institutes of Health.

Several reports on pulmonary hypertension were published from this registry in the 1980s, highlighting the severity of this condition and revealing the extent to which pulmonary hypertension can be the consequence of multiple diseases, including immune vascular diseases, HIV infection, and portal hypertension.

The registry also provided valuable information, including the first survival equation whereby haemodynamic data, such as cardiac index, right atrial pressure, and mean pulmonary artery pressure, were relevant data in terms of survival^47,48^. These reports and the second epidemic of pulmonary hypertension with another popular appetite suppressant medication dexfenfluramine-phentermine “fen-phen” in the 1990s (see ‘Appendix’) renewed interest in this condition among the medical community.

The fourth milestone in the history of pulmonary hypertension was the introduction of a specific therapy for primary pulmonary hypertension. Epoprostenol, a synthetic salt of prostacyclin given via a continuous intravenous route, was shown in 1996 to reduce mortality^50^. These encouraging findings, the fen-phen epidemic, and the 25^th^ anniversary of the first meeting in Geneva, prompted many in the field to convene the second WHO Symposium of pulmonary hypertension in Evian in 1998.

**Figure 1.**
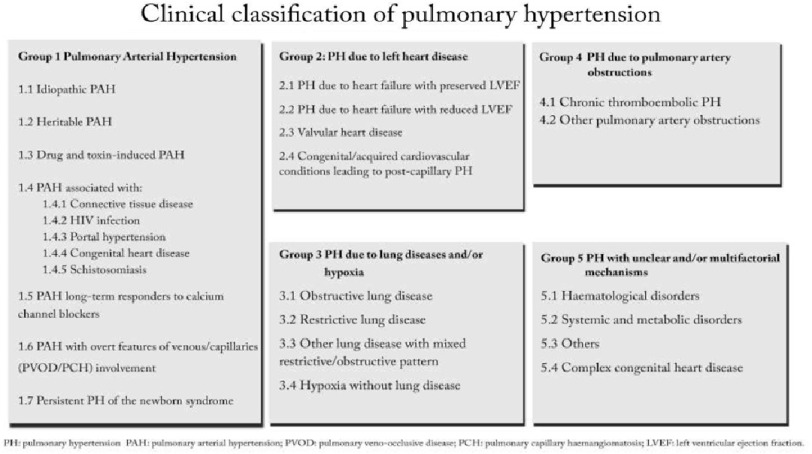
Current classification of PH as per the 6^**th**^ WHPH in Nice 2018^49^.

**Figure 2.**
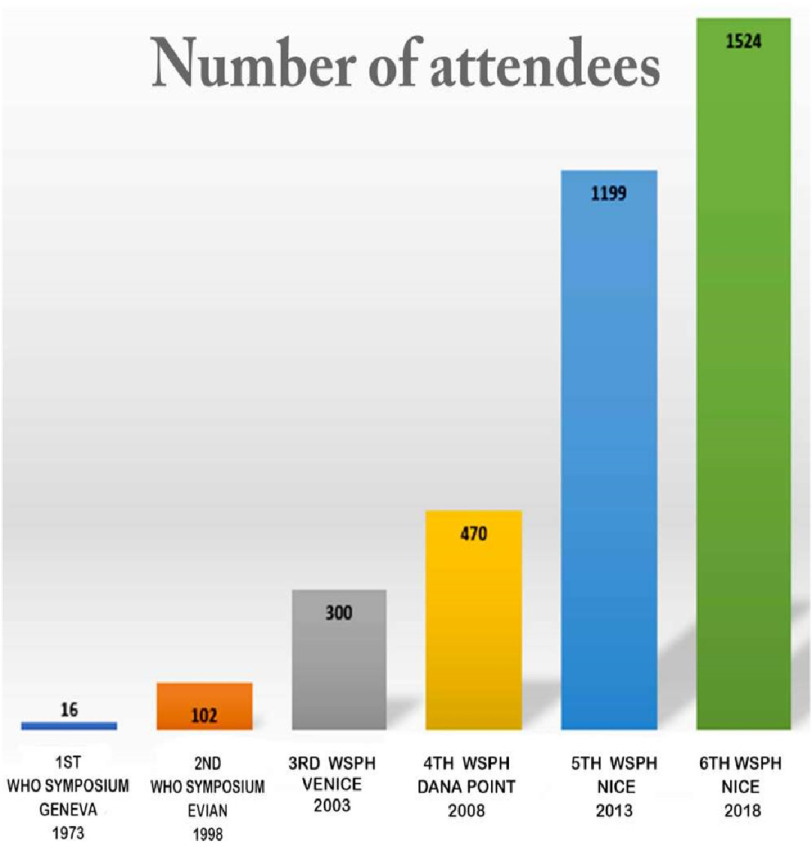
Attendees of the World Symposia on Pulmonary Hypertension.

At this meeting, a new diagnostic classification of pulmonary hypertension was proposed (Figure 1), which is still in use today, albeit with continuous revision. Subsequently, meetings were held regularly every five years and were collectively known as the “World Symposium on Pulmonary Hypertension” (WSPH). The 3rd WHPH was held in 2003 Venice^51^, the 4th WHPH in 2008 Dana Point, California, the 5^th^ WHPH was Nice, France in 2013^52^, and the 6^th^ WHPH was again in Nice 2018^49^. The 7th WHPH in 2023 is planned to take place in Orlando, Florida, USA. These meetings have become the cornerstone for the global management of pulmonary hypertension and have increasing attendance every 5 years (Figure 2).

The introduction of pulmonary hypertension specific therapy over the last 20 years has significantly contributed to the management of this condition. After the introduction of intravenous epoprostenol (Flolan^®^), the first oral therapy for pulmonary hypertension was the dual endothelin receptor antagonist Bosentan, which was approved in 2001 for the treatment of advanced disease pulmonary arterial hypertension. It was followed by the approval of sildenafil, a phosphodiesterase type 5 inhibitor, in 2005.

Other medications were mainly prostaglandin analogues such us treprostinil (Remodulin^®^) in 2002, with enough chemical stability to be administered at ambient temperature. Iloprost (Ventavis^®^), in intravenous, oral or inhaled formulations, was approved by the FDA in 2004.

Oral prostaglandin analogues, like beraprost, were used in Japan and Korea, but not approved elsewhere. In 2016, selexipag (Uptravi^®^), an oral prostacyclin-receptor agonist, was licensed for pulmonary arterial hypertension therapy. Another endothelin receptor antagonist, ambrisentan (Letairis^®^ in the USA; Volibris^®^ in Europe) was approved in 2007. Macitentan (Opsumit^®^) was approved in 2013. Others phosphodiesterase type 5 inhibitors were also licensed for pulmonary hypertension, such as tadalafil (Cialis^®^) in 2009.

Riociguat (Adempas^®^), which works by enhancing the level of cGMP via activation soluble guanylate cyclase enzyme, was approved in 2013 for both pulmonary arterial hypertension and chronic thromboembolic pulmonary hypertension.

These developments were associated with a significant increase in research activities in the pathogenesis and management of pulmonary hypertension globally. It is also noticeable that there has been a substantial increase in the number of research papers and presentations at international meetings over the last 20 years.

There have also been many patient registries and specialized patient databases, in addition to clinical trials and the development of various guidelines in many continents. This enhanced awareness among the medical profession was complemented with the increasing number of pulmonary hypertension patient advocacy organizations on a global level. These were accompanied by interest groups in various cardiology and respiratory societies and the establishment of global independent organizations like the Pulmonary Vascular Research Institute (PVRI) and others.

## The epidemiology of pulmonary hypertension

Many factors influence the epidemiological assessment of this syndrome. The exact incidence (patients per million who have just received a diagnosis) and prevalence (patients per million who have previously received a diagnosis and are in a follow-up program) of pulmonary hypertension in the world is not known and can be challenging to evaluate.

The first cases were discovered via pathological assessment. Cardiac catheterization, and later widespread screening tools such as echocardiography, Doppler echocardiography, and other modalities of advanced imaging techniques, lead to a steady increase in clinical diagnoses.

Furthermore, the recent introduction of the specific therapy of pulmonary hypertension and increased marketing boosted interest in the diagnosis and enhanced clinical awareness of this syndrome. These factors lead to more cases being diagnosed, increasing the incidence of pulmonary hypertension worldwide^5,53–58^.

Pulmonary hypertension is not one “specific” disease. Unlike systemic hypertension, most of the pathology is due to genetic, environmental, and other systemic diseases. “Primary” (later labelled “idiopathic”), where the causality is unknown, is probably one of the rarest forms of pulmonary hypertension.

The current classification of pulmonary hypertension was adopted during the 1988 Second World symposium in Evian^59–61^, and subsequently modified in succeeding symposia. The continuous revision of the classification adds to the complexity of defining the epidemiology of pulmonary hypertension globally.

The current classification of pulmonary hypertension is rather simplistic. More granular identification, based on individual patient phenotypes to increase the homogeneity of patients and management approach^57,62,63^, might improve the management of the disease. This will have a significant impact on redefining the patients’ demography, increasing the complexity of the epidemiological picture of pulmonary hypertension in general^3^. Furthermore, the regional variation and the poor reporting of pulmonary hypertension in many developing countries added to the challenge of assessing the global epidemiology^4,64,65^.

The potential changes in the clinical definition of pulmonary hypertension^46,66,67^ can also contribute to these difficulties. In 2018, the 6th WSPH proposed to reconsider the haemodynamic definition of pulmonary hypertension as when mPAP >20 mmHg and vascular resistance (PVR) ≥ 3 WU^68^. If this definition applied globally, it will eventually change the epidemiological assessment of this condition further and may increase the incidence of pulmonary hypertension globally.

**Figure 3.**
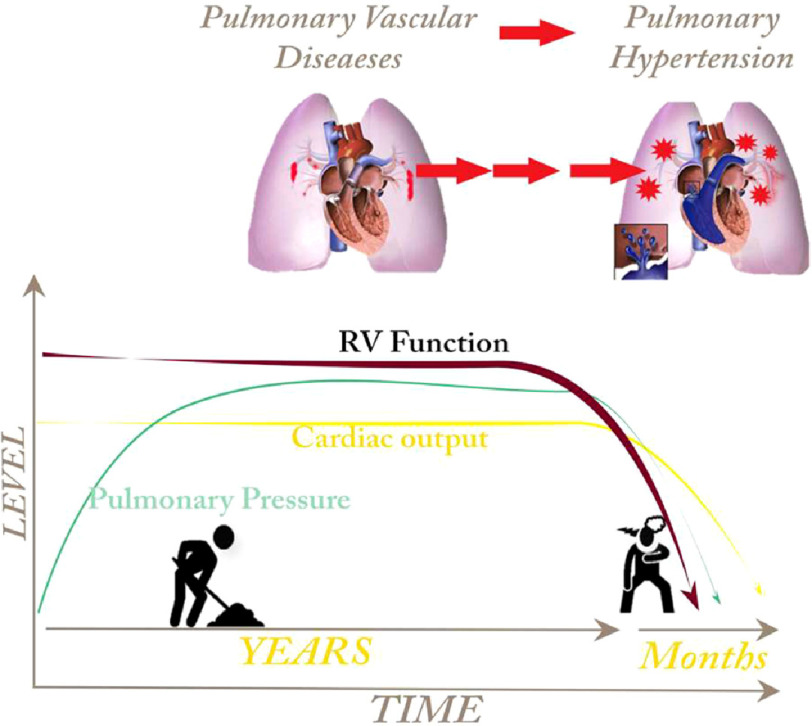
Progress of pulmonary vascular diseases.

Most of the current registries suggest that the diagnosis of pulmonary hypertension is usually delayed, not only because of inappropriate differential diagnosis, but because the pathology may exist for years without symptoms. Thus, many patients will have some pulmonary vascular pathology that, with time, will progress. The symptoms and clinical presentations of pulmonary hypertension will appear later when the increase of pulmonary vascular resistance and pulmonary pressure cause strain of the right ventricle^69,70^ (Figure 3). Therefore, the epidemiology of “pulmonary vascular diseases” may be much higher than “pulmonary hypertension” as a clinical presentation.

This means that the epidemiology of pulmonary hypertension should be considered as ‘dynamic’ and in need of continuous evaluation at any time and geographical location.

Currently, registries are the primary tools used to estimate the Incidence and prevalence of pulmonary hypertension. Since the first NIH pulmonary hypertension registry in the USA, more than twenty registries all over the world were planned^47,48^. There were local, hospital-specific, or larger national registries from France^71^, Scotland^72^, United Kingdom^57^, Australia^73^, Spain^74^, China^75^, Germany and other European centres^76,77^, Japan^78^, Korea^79^, Czech Republic^80^, Brazil^81^, Switzerland^82^, Holland^83,84^, Portugal^85^, Greece^6^, Algeria^86^, Saudi Arabia^87,88^, Israel^89^, India^90^ and the USA^91–93^.

These registries were supplemented by many other smaller regional registries in middle- to low-Income regions^94^. Collectively, they collected baseline characteristics and clinical outcome data on more than 10,000 patients, with the main emphasis on Group 1^95^. Most of these registries used their data to estimate the distribution of various aetiologies and the general Incidence and prevalence, which is not an adequate epidemiological method^96^.

These registries showed a progressive increase in the incidence and prevalence of pulmonary arterial hypertension over the years. In the 1990s, the NIH registry suggested the incidence of primary pulmonary hypertension (later redefined as idiopathic) should be increased from 1 to 2 cases per million people in the general population^97–100^. It was previously underestimated as many of these patients might have had a genetic predisposition.

Families with pulmonary hypertension were first described in 1951^38^, but more families were recorded by 1984^101^. In the NIH registry, familial primary pulmonary hypertension accounted for 6 per cent of all cases^47,102^. The current registries, for the last twenty years, indicate that the incidence of pulmonary arterial hypertension ranges from 2 to 10 cases per million adults per year, and its prevalence varies from 11 to 60 cases per million adults^103–105^.

The French registry in the early 2000s, covering 17 university hospitals in France, included 674 consecutive patients over 18 years of age. It reported that idiopathic cases represented 39.2%, and familial, 3.9% of the study population. The remainder were pulmonary arterial hypertension associated with other conditions.

These investigators estimated the prevalence of pulmonary arterial hypertension in France as 15 cases per million, and the incidence as 2.4 cases per million of the adult population per year^71^.

The registry from Scotland^72^ of 374 patients, aged 16–65 years, hospitalized with pulmonary arterial hypertension between 1986–2001, found the prevalence of pulmonary arterial hypertension was 52 cases per million population, and the annual Incidence was 7.1 cases per million population^57,72^.

Data recorded from eight pulmonary hypertension centres identified 482 newly diagnosed patients in the United Kingdom from 2001 to 2009. The data suggested a spread of 93% idiopathic, 5% heritable, and 2% anorexigenic-associated pulmonary arterial hypertension. The estimated incidence was 1.1 patients per million per year, and the prevalence was 6.6 patient cases per million^57^.

The Spanish Registry of Pulmonary Arterial Hypertension estimated prevalence was 16 patients per million of population and the estimated incidence was 3.7 per millions^74^.

The REVEAL Registry is the largest USA registry of 2,525 adults meeting traditional hemodynamic criteria. It showed that 46.2% were idiopathic, familial was 2.7%, and the remainder due to other etiologies of pulmonary arterial hypertension^14,15^.

Other registries showed more variation. For example, a nationwide epidemiological survey in Japan in 2012 included 1377 patients (new: 389, recurrent: 988) and suggested the prevalence to be 15.6 per million. Idiopathic and heritable pulmonary arterial hypertension were the most common type of PAH (66.6%) and pulmonary arterial hypertension associated with congenital heart disease (16.3%), and pulmonary arterial hypertension associated with connective tissue diseases, were common (11.1%)^106^.

In Algeria^107^, one registry prospectively collected data from 120 patients with pulmonary arterial hypertension between 2009 to 2014 and reported 30% of patients had idiopathic pulmonary arterial hypertension, 5.8% familial pulmonary arterial hypertension,11.6% had connective tissue disease, and 26.6% congenital heart diseases.

The NIH registry showed predominant female to male (1.7:1)^102^. Other registries also showed the predominance of female to male pattern, especially in the younger age groups^14,15,57,72,74,107^. For example, the French registry was (1.9:1)^71^ and in the COMPERA Study was 2.3:1^76^. However, the REVEAL Registry found a higher proportion of women (4.1:1) whereas the gender ratio in elderly patients (median age, 75 years) was almost even (1.2:1)^76^.

The mean age of patients with primary (idiopathic) pulmonary hypertension in the NIH registry was 36 years. Registries in the last twenty years showed more older patients between 50 and 65 years diagnosed with pulmonary arterial hypertension. The REVEAL registry median ages were 50-53 years and were similar between males and females^57,72,76,92,93^, whereas the COMPERA registry the median age at diagnosis was 71 years^76^.

Studies in Japan noticed the average age of their cohort was 53.0 ±19.4 in 2012, older than the previous survey in 2004 (41.9 ± 19.5) and the ratio of elderly patients (older than 65 years) was 33.3% in 2012 but 18.3% in 2004^106^. Many studies suggested that with age, the disease may show different phenotype and survival rates. This subject still needs further investigation.

## Tools, methods, and global estimates

The developing world, where 7 billion people live, paid less attention to the diagnosis of pulmonary hypertension and the pattern and etiologies differ from those in developed countries^64,108^. It is difficult to estimate the real extent of pulmonary hypertension in developing countries due to the lack of proper epidemiological investigations and patient-based studies.

This review adopts a simple method to assess the global impact of pulmonary vascular diseases in the world, by determining the proportion of patients who are likely to develop pulmonary vascular diseases due to various clinical conditions from the published data. This approach can be problematic, may be controversial and is expected to be subjected to some guesswork. It is, however, the only method currently available, due to the scarcity of hard data. It is hoped that future studies will be able to confirm or refute the picture portrayed by our estimate.

## Pulmonary arterial hypertension

There an estimated 40,000-100,000 patients globally with pulmonary arterial hypertension, excluding patients with infectious diseases^4^. This is likely an underestimate because many patients in the developing world in this group have not been treated or diagnosed yet.

## Infectious diseases

We believe that infectious diseases are among the most significant contributors to pulmonary vascular disorders. A wide-ranging variety of infectious diseases can contribute to the causation of pulmonary vascular diseases and consequently, pulmonary hypertension, especially in the developing world.

## Helminthic diseases

Schistosomiasis affects over 200 million people worldwide and is the third most common parasitic disease after malaria and amoebas. It is endemic in 74 countries, including Africa, Brazil, the Middle East and Southeast Asia^109–111^ and is well documented in causing pulmonary vascular diseases. Recent observations suggest some difficulties in complete eradication of the disease despite the availability of anti-helminthic agents and other public health measures^111–114^.

There are different species of schistosomiasis, the most common to affect humans are *S. mansoni* ,*S. haematobium,* and *S. Japonicum*. Chronic schistosomiasis is the most prevalent form of the disease in regions endemic for schistosomiasis, due to repeated exposure and re-infection^115,116^. The cardinal pathological component of the of schistosomiasis is not the mature worm, which has evolved immune evasion mechanisms that allow them to remain incognito within the bloodstream, but by the highly antigenic egg-associated pathology that is central to the morbidity and mortality^115^. Liver disease develops secondary to entrapment of eggs in portal venules and is initially presinusoidal, resulting in periportal fibrosis, and the development of “Symmers pipe stem fibrosis”^115,117^.

Approximately 5–10% of the patients chronically infected with schistosomiasis develop the hepatosplenic form of the disease, with hepatomegaly, splenomegaly, portal hypertension with esophageal varices and portocaval shunting which facilitate a route for the eggs to enter the lung.

The presence of schistosoma eggs in the lungs of African natives was described as early as 1885^118^, which was followed by several reports from Egypt in the first half of the 20th century describing pathological and clinical manifestations of pulmonary vascular diseases. In the second half of the 20th century, reports of many small series appeared in the literature, this time mainly from Brazil and occasionally a few cases from Africa, and more recently from China.

The highly antigenic eggs develop an immunological reaction that leads to the development of granuloma, and subsequent remodeling of pulmonary arterioles. Severe intimal, medial, and adventitial hypertrophy and a proliferation of inflammatory cells occurs in the pulmonary vasculature, which contributes to the development of pulmonary hypertension^115,119–122^.

In earlier studies, the prevalence of pulmonary vascular pathology ranged from 7.7% to 33%, mostly based on pathological assessment^123–127^. The most recently available data of pulmonary hypertension patients have come from Brazil, which has a good schistosomiasis control program, and its clinical care is far superior to many developing countries. Their data estimated that 7.7% to 10.7% of patients may have some form of pulmonary hypertension secondary to schistosomiasis^115,124,125^; but these estimates were based on case control studies rather a proper epidemiological evaluation.

Epidemiological studies may be difficult to conduct widely as there are no specific tests or biomarkers to diagnose schistosomiasis-induced pulmonary vascular pathology. However, the situation may be different in Africa, where 80% of global schistosomiasis patients are; and the continent as whole has far worse disease control than Brazil. In addition, many other comorbidities complicate the picture in Africa.

We noticed from our experimental observations in our lab that the changes in the pulmonary vasculature after schistosoma infection are far more common. It was found in 46% of experimental animals infected with schistosomiasis, but only 12% had evidence of right ventricular hypertrophy, which reflects significant pulmonary hypertension.

These experimental and clinical data reviewed above suggest that some pulmonary vascular diseases can be found in almost half of patients with hepatosplenic disease secondary to schistosomiasis, thus we can conservatively estimate that 20-50 million people may have this pathology worldwide and the clinical presentation of pulmonary hypertension can be expected to be around 4–16 million patients worldwide^65,122^. This makes schistosomiasis the most prevalent causes of Group 1 of the current classification (pulmonary arterial hypertension^122^.

Many other helminthic diseases can induce pulmonary hypertension, such as *Wuchereria bancrofti*, a threadlike worm that causes filariasis (elephantiasis)^128,129^. *Clonorchis sinensis* (Chinese liver fluke) is a widespread parasite in southeast Asia and has been associated with cases of pulmonary hypertension^130^. Some investigators have reported that other parasitic diseases, such as hydatid cysts, can induce pulmonary hypertension^131^. However, these are a small number of case reports, thus its epidemiological profile is far from being clear.

## Viral infection

Viral infections, such as human herpes virus-8, and HIV, showed evidence of pulmonary vascular pathology^132,133^. The UNAIDS program estimated up to 44 million people were living with HIV by the end of 2018, with the highest prevalence in sub-Saharan Africa^134^. HIV can increase the risk of developing pulmonary hypertension by up to x 2000.

Careful analysis of the published data suggests that the estimated prevalence of HIV-pulmonary hypertension ranges from 0.4 to 11.5% globally^135,136^. The true prevalence of clinical pulmonary hypertension in HIV patients is likely to vary according to regional differences because of differences in environmental, cultural, and genetic factors^135^. The condition can be exacerbated by using addictive drugs found in many herbal remedies in the developing regions^137^. Furthermore, co-exposure with more than one infection can be an issue. In some parts of Africa, over 50% of patients infected with HIV are co-infected with schistosomiasis^135^.

Taking into consideration the number of patients who have HIV worldwide, we estimate about 170,000 to 1 million patients suffer from pulmonary hypertension secondary to HIV.

## Other infections

Some bacterial infections, such as *B. Pertussis*, may trigger pulmonary hypertension^138,139^. Other bacterial infections that cause granulomatous reactions in the lungs, like tuberculosis, have been suspected, but not thoroughly evaluated. Indeed, recent cases and communications from Africa and India suggest the potential role of tuberculosis in PVD^140–142^. Recent initial reports indicate that fungal infections, like *P. brasiliensis,* which causes paracoccidioidomycosis in Brazil, can cause PVD in patients and lab animals^143^. However, these are small observation and do not allow a proper epidemiological estimate for these infections.

## Haemoglobinopathies

Hemoglobinopathies are the most common genetic defect globally. Sickle cell anaemia is the most common monogenic disorder. Each year about 300,000 infants are born with significant haemoglobin disorders, including more than 200,000 cases of sickle-cell anaemia in Africa alone^144^. Chronic hemolytic anaemia has increasingly been identified as a risk factor for the development of pulmonary hypertension. It is also associated with chronic organ damage, especially kidney and heart^145,146^, The prevalence of pulmonary hypertension in sickle cell disease patients was variable in a population, depending on other comorbidities. It ranges from 6% to 12.9%, but can be higher in some populations in Africa^147–149^. The UN estimates that there are between 20 and 25 million people worldwide living with sickle cell anaemia, of which 12–15 million live in Africa alone^150^. Thus, we estimate globally, 2 to 4 million patients suffering from pulmonary hypertension second to sickle anaemia.

Thalassemia is another form of hemoglobinopathy which is common in Mediterranean countries, can cause pulmonary hypertension, and can be associated with the development of other organs. The prevalence of pulmonary hypertension was estimated at 1.1 to 4.2%^151,152^, which differs among different forms of thalassemia (thalassemia α-, β-, and intermedia). In 2015, about 280 million people had thalassemia, with around 439,000 having the severe disease^153^. Therefore, an estimated 2 to 12 million people may have some sort of pulmonary hypertension due to thalassemia.

## High altitude

Nearly 140 million people live in high- altitude locations worldwide^154–157^ and are therefore at risk of developing pulmonary hypertension. The pattern of pulmonary hypertension seen in these locations varies from one region to another, probably because of some genetic variation as well as an underlying congenital cardiac condition like the presence of patent ductus arteriosus^158–160^. Generally, it is estimated that around 5% to 18% of patients in these locations may have clinically-significant pulmonary vascular pathology that may cause clinically-significant pulmonary hypertension^158–160^. This means an estimate of 7 to 25 million high-altitude inhabitants may suffer from pulmonary hypertension.

## Respiratory disorders

Chronic obstructive pulmonary disease (COPD) is the third most frequent cause of morbidity and mortality worldwide. COPD is a chronic and progressive disease with most cases as consequence of chronic bronchitis, smoking, air pollution and other environmental factors^161,162^. Pathologically, COPD causes major impairments in all lung compartments with small airways, and pulmonary vasculature^163–168^.

Pulmonary hypertension is a common in COPD patients, but there is variability in the estimated prevalence, because of differences in the definition of pulmonary hypertension in various studies^169^. Furthermore, the prevalence estimate may be inflated due to the over-representation of patients with advanced disease stage^169^. Various studies have reported that about 5% to 50% of patients with COPD may have pulmonary artery pressures over 25 mm Hg^167,170,171^.

The Global Burden of Disease Study reports a prevalence of 251 million cases of COPD globally in 2016 with 3.17 million deaths (90% of COPD deaths occur in low”- and middle-income countries)^172^. Thus, the estimated number of patients with pulmonary hypertension secondary to COPD can reach 25 to 100 million worldwide.

## Cardiac conditions

Although rheumatic heart diseases (RHDs) are decreasing, they are still a part of any cardiology practice in the developing world^173–175^. The prevalence of RHD varied widely depending mainly on the level of the country’s income. The main damage is to the cardiac valves, most commonly the mitral valve, and to a lesser extent, the aortic valve.

Pulmonary hypertension is a well-recognized sequalae of RHD. It is found in 10–70% of patients, according to the pathological severity of mitral valve disease, and in up to 65% of those with symptomatic aortic stenosis^176,177^. The degree of pulmonary hypertension was an independent predictor of prognosis^65,178^ and carries substantial clinical risk, even post-operatively. Fortunately, pulmonary hypertension is a curable and reversible condition, particularly with valve replacement or valvuloplasty. It is estimated that RHDs affect 33.4 million people globally, and cause 319,400 deaths^179–182^. Thus, we estimate that up to 3 to 10 million patients may have some form of pulmonary vascular complications secondary to rheumatic heart diseases.

Similarly, congenital heart diseases contribute to the development of pulmonary vascular diseases. CONCOR study found that pulmonary hypertension was present in 4.2% of cases^183^, although this varies according to the cardiac lesions and the outcome of surgical intervention^184,185^. If we consider that 24.5 million people are affected by congenital heart disease worldwide, we can expect about 1 million patients to be suffering from pulmonary hypertension secondary to congenital heart disease.

Heart failure is a growing public health problem worldwide, affecting at least 26 to 61 million people worldwide, and increases dramatically with an ageing population^186,187^. Its prevalence shows geographic variations^188^. Preliminary data suggested heart failure prevalence is higher in Asia-Pacific compared to Western countries^82,83,189^.

Pulmonary hypertension is a frequent complication of heart failure and contributed to the worse prognosis^190^. It occurs in both heart failure with reduced LV ejection fraction (HFrEF) and heart failure with preserved LV ejection fraction (HFpEF). Pulmonary hypertension is common with the later phenotype^191,192^. In general, the prevalence of pulmonary hypertension, as assessed by right heart catheterization, was between 33% and 68% of heart failure. HFpEF showed higher prevalence from 50% to 80%^193–195^. Therefore, heart failure is the most common aetiology of pulmonary hypertension, particularly in the developing world, and thus contributes significantly to pulmonary vascular diseases globally^4,65^.

## Chronic thromboembolic pulmonary hypertension (CTEPH)

CTEPH is a rare and progressive pulmonary vascular disease, classified as Group 4 according to the current classification. Its global epidemiology is limited. Recently there has been an increase in incidence and prevalence due to more awareness, accurate diagnosis, and the introduction of specific therapy for this condition^196^.

Acute pulmonary embolism, mainly due to venous thrombosis, is the most common aetiology of this form of pulmonary hypertension. Recent data suggested that about 4% of pulmonary embolism patients will develop CTEPH within two years^197^. The average estimated incidence in the USA is 66–104 and in Europe 3–5 cases per 100, 000 population, while in Japan these rates were lower at 6.7 and 1.9 per 100, 000 population^198^.

Other investigators suggested that the general estimate of CTEPH per one million people in the general population is 3–30 patients^199^. There is significant variation globally due to many factors, including under-diagnosis of this condition and a lack of public awareness of pulmonary embolism in general^200,201^.

The incidence of pulmonary embolism is estimated to be approximately 60 to 70 per 100,000, and venous thrombosis 124 per 100,000 of the general population^202,203^. Taking in consideration about 600 to 1,000 cases per million of pulmonary embolism per year, and the incidence of CTEPH is about 4% as mentioned above, leads us to suggest that the approximate prevalence of 250,000 to 500,000 of CTEPH globally.

## In summary

The discussion above suggested that pulmonary hypertension is a global disease, thanks to the diversity of aetiologies^4,204^. Chronic cardiac diseases (group 2) and chronic respiratory diseases (group 3) are the leading cause of pulmonary hypertension globally, but no specific therapies are available presently, unlike group 1 (pulmonary arterial hypertension).

Infectious diseases, mainly Helminthic diseases, represent the most common cause of pulmonary arterial hypertension globally. The prevalence of Group 4 is on the increase due to the availability of specific medication (Riociguate^®^) and to the surgical intervention progress.

**Figure 4.**
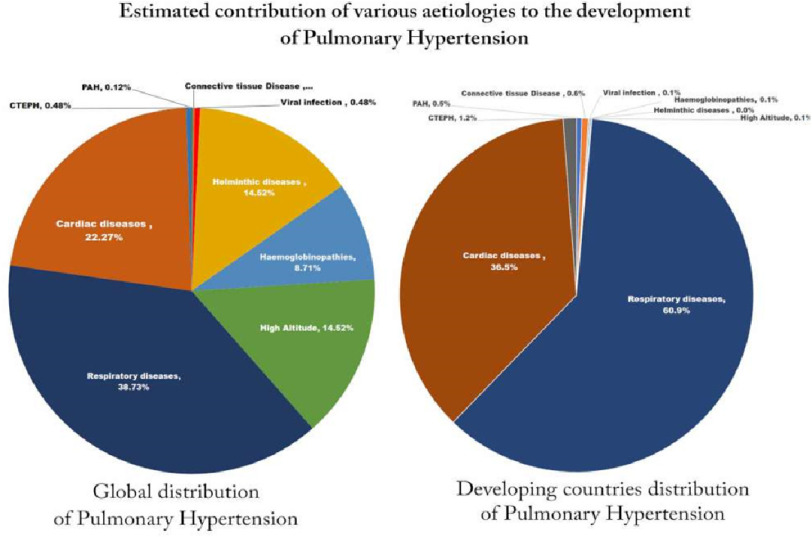
The estimated distribution of various forms of pulmonary hypertension the developing and developed countries.

The distribution of pulmonary vascular diseases and pulmonary hypertension in the developed world shows a very different picture compared to the developing world (Figure 4). It is estimated that 80% of patients with pulmonary hypertension live in the developing world^4,94^. The population in the developing world is about 7 billion vs 1 billion in the developed world. Our speculative calculations and accounting for population numbers, the likelihood to develop pulmonary hypertension is twice than those living in the developed countries^94^.

The historical development of the increase in medical knowledge of pulmonary hypertension and the development of a recent epidemic (‘Appendix’), in addition to the introduction of specific therapies, has enhanced interest and awareness and lead to an increase in the diagnosis and management of more patients with pulmonary hypertension^4^. Therefore, the notion that this condition to be considered orphan diseases should be reconsidered.

The orphan disease definition is country-specific and subject to regional variation^108^. It is rather challenging to define the status of “orphan” on the basis of the number of patients globally. Thus, collectively, pulmonary hypertension cannot be considered an orphan condition from a global perspective.

Redefining the condition due to their various etiologies, some forms of pulmonary hypertension are much rarer and can be defined as “orphan” conditions, for example, familial pulmonary hypertension or in following the current trend of deep phenotyping.^1^ 1See the article in this issue by Paul A Corris: The importance of deep phenotyping PH registries with a focus on the PVRI-GoDeep registry

## Appendix. Pulmonary hypertension epidemics

There was an unexpected increase in the number of patients with pulmonary hypertension in the last four decades of the 20th century. It was immediately associated with the extensive use of drugs to treat obesity and other substances. They were labelled as “epidemics” because of the substantial and sudden increase. These events have influenced many aspects of our understanding and pathogenesis of pulmonary hypertension as well as medical, pharmaceutical, and medico-legal aspects^205^.

## Aminorex epidemic

Between 1967 and 1972, a sudden 10-20x increase (about 2,000 cases per 1 million) in the incidence of pulmonary hypertension, with four times as many women as men, was noticed in Austria, Germany and Switzerland^205,206^. This increase was associated with the intake of the appetite suppressing drug aminorex (2-amino-5-phenyl-2-oxazoline) (Menocil^®^), which was approved in 1965 in these countries for over-the-counter sale^207,208^. The clinical presentation of pulmonary hypertension started 6–12 months after starting the medication^209^. This sudden significant increase in pulmonary hypertension led to the withdrawal of the drug from the market in 1968. Still, more cases were reported for several years till 1972 when epidemic ended^205^, suggesting that this effect can be chronic but is potentially reversible^205^.

The experience with aminorex in obesity treatment brings into light the potential association of obesity drugs (and probably other drugs or substances) with pulmonary hypertension. This first epidemic led to more interest in pulmonary hypertension and was one of the drivers for the WHO to convene the meeting on pulmonary hypertension in Geneva.

## Fenfluramine epidemics

Fenfluramine (Pondimin^®^) acts as a serotonin releasing agent which was introduced in the 1970s to Europe and the USA. It was not a great success as it reduced weight temporarily and was associated with uncomfortable side effects. In the 1980s, the addition phentermine showed a dramatic clinical impact with less side effect^210^. This combination was called (Fen-Phen^®^).

Physicians started to prescribe it off-label and become a sensational drug in the media in the 1990s. This success encouraged some pharmaceutical companies, after the expiration of fenfluramine patent, to develop its d-isomer, dexfenfluramine (Redux^®^).

During this time, the preliminary results from the International Primary Pulmonary Hypertension Study (IPPHS)^211^, case-control epidemiological studies conducted in five European countries over two years, became known. It supported an earlier report in the 1980s^212,213^ that there was over 10 times more risk of developing pulmonary hypertension when these drugs were used for more than 3–6 months^100,214^.

Nine (22.5%) out of 40 patients evaluated resulted positive for the presence of germline bone morphogenetic protein receptor (BMPR) type 2 mutations. In these patients, the duration of exposure to fenfluramine was significantly lower than in patients without mutations^215^. Despite these findings and concern in the medical community, and after the intense debate, the FDA approved (Redux^®^) on the 29th of April 1996, without a black box warning.

A substantial marketing advertisement campaign followed, and the sale of the drug rocketed quickly. Within a year, and in 1997 reports of pulmonary hypertension and heart-valve abnormalities surfaced^11–14,215^. The FDA withdraw the market authorization on the 15th of September 1997^15–18^.

It was quickly followed on the 7th of October 1997 by intense and perplexing legal actions by thousands of patients against the pharmaceutical companies which lasted for more than 15 years. How many people were harmed by these drugs is still far from being known. Some reports suggested more than 30,000 lawsuit claims for compensation and damages were filed. The legal actions cost the concerned pharmaceutical companies billions of dollars in compensation.

## Rapeseed oil epidemics

When rapeseed cooking oil was legally marketed in Spain in 1981, more than 20,000 cases were reported in, and many deaths were related to pulmonary hypertension with the pathological changes of perivascular inflammatory infiltrates resembling pulmonary veno-occlusive disease^216^. This was followed by another epidemic in the second half of 1980s^217^.

## L-tryptophan epidemics

In 1989 in New Mexico an about 1,400 cases of diffuse myalgia, eosinophilia and pulmonary hypertension were noticed to be associated with L-tryptophan prescribed over the counter^218–221^.

## Other illicit drugs

Illicit drugs have been implicated with a higher incidence of pulmonary hypertension. For example, heroin (77.9%), cocaine (46.8%) and amphetamines (18.2%), especially if it is coupled with other conditions or risk factors like HIV or portal hypertension^213,222^.
